# Image quality evaluation of ultrasound imaging systems: advanced B‐modes

**DOI:** 10.1002/acm2.12544

**Published:** 2019-03-12

**Authors:** Elisabetta Sassaroli, Calum Crake, Andrea Scorza, Don‐Soo Kim, Mi‐Ae Park

**Affiliations:** ^1^ Department of Radiology Brigham and Women's Hospital & Harvard Medical School Boston MA USA; ^2^ OxSonics Ltd The Magdalen Centre Oxford UK; ^3^ Department of Engineering Roma Tre University Rome Italy; ^4^ Department of Radiology Boston Children's Hospital Boston MA USA

**Keywords:** adaptive speckle reduction, B mode, compound imaging, harmonic imaging, speed of sound correction, tissue aberration correction, ultrasound imaging quality assurance

## Abstract

The Quality assurance of ultrasound clinical imaging systems is essential for maintaining their performance to the highest level and for complying with the requirements by various regulatory and accrediting agencies. Although there is no standardization yet, most of the quality assessment procedures available in literature are proposed for B‐mode and Doppler imaging. However, ultrasound imaging systems offer a variety of advanced imaging modes, besides B‐mode and Doppler, which are primarily aimed at improving image quality. This study presents computer‐based methods for evaluating image quality for the advanced imaging modes of ultrasound imaging systems: harmonic imaging, spatial compounding imaging, adaptive speckle reduction, and tissue aberration correction. The functions and parameters proposed for evaluating image quality are: grayscale mapping function, image contrast, contrast‐to‐noise ratio (CNR), and high‐contrast spatial resolution. We present our computer‐based methods for evaluating image quality of these modes with a number of probe and scanner combinations, which were employed to image targets in ultrasound phantoms. The functions and parameters here proposed in image quality performance evaluation are: grayscale mapping function, image contrast, CNR, and high‐contrast spatial resolution. We show that these quantities could be useful in developing standardized methods for evaluating the advanced ultrasound imaging modes, especially when the advanced mode resulted in subtle visual differences.

## 1 INTRODUCTION

Performance evaluation or quality assurance (QA) of ultrasound (US) equipment is necessary, as for any other medical imaging equipment, for ensuring the safety of the patient and operator, for maintaining the image quality as specified by the manufacturer's recommendations, and for complying with the requirements by various regulatory and accrediting agencies. An effective QA protocol makes it possible to detect a fault or a change in performance at an early stage so that appropriate technical help can be requested. Currently, QA of US imaging systems is usually performed in B‐mode and Doppler imaging. Although there is no worldwide standardization yet, tests suitable for B‐mode imaging are well documented in the literature^1^, ^2^, ^3^, ^4^, ^5^, ^6^, ^7^, ^8^, ^9^, ^10^, ^11^, ^12^, ^13^, ^14^, ^15^, ^16^ and they have been published by a number of professional organizations: AIUM (American Institute of Ultrasound in Medicine),^1^, ^2^, ^3^ American Association of Physicists in Medicine (AAPM),^4^ American College of Radiology (ACR),^5^ European Federation of Societies for Ultrasound in Medicine and Biology (EFSUMB),^6^ and Institute of Physics and Engineering (IPEM).^7^ These tests usually include: visual inspection of the components of the US system (scanner/probes), display monitor performance, image uniformity, sensitivity (maximum depth of visualization, signal‐to‐noise ratio), geometric accuracy, spatial resolution, and contrast resolution. In USA, QA of personnel qualifications and equipment consent hospitals to gain accreditation or re‐accreditation of their practices by an accrediting agency (e.g., ACR, AIUM). In addition to B‐mode and Doppler imaging, US imaging systems offer now a variety of advanced imaging techniques which are primarily aimed at improving image quality. These techniques include: harmonic imaging (HI),^17^, ^18^, ^19^, ^20^, ^21^ spatial compound imaging (SCI),^21^, ^22^, ^23^, ^24^ adaptive speckle reduction (SR),^25^, ^26^ and tissue aberration correction (TAC),^26^, ^27^ also known as speed‐of‐sound correction. Emerging clinical imaging modes include: elastography^28^ and 3D/4D imaging.^29^

For the accreditation process, no specific QA tests are currently required for Doppler imaging and for the advanced and emerging imaging modes. Therefore, it is not surprising that these tests are not normally performed in a routine QA of medical US equipment. The difficulty in their implementation, likely due to a lack of scientific literature and to the high costs, is followed by lack of norms, standardization, and accreditation programs. Nevertheless, since the US technology is widespread, there is a great need to develop effective and reproducible QA tests also for the advanced modes and emerging imaging modes.

**Figure 1:**
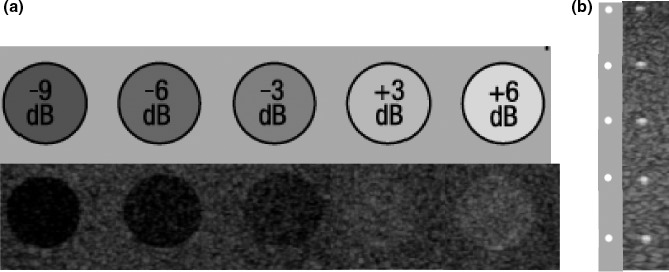
(a) Target of known nominal contrast in phantom. (b) Nylon filaments of known nominal size (0.1 mm) in phantom.

In a previous paper,^14^ we discussed our computer‐based QA tests for basic B‐mode imaging. In this paper, we present examples of computer‐based QA implementation for some US advanced modes. Since the advanced modes are aimed at improving image quality, image contrast, and high‐contrast spatial resolution (HCSR) are usually evaluated for these modes. The image contrast test is aimed at establishing the ability of the US imaging system to detect subtle differences in the echogenicity of two targets. Image contrast is usually assessed using targets of known nominal contrast in a phantom [Fig. 1(a)] at fixed ultrasound settings and is affected by both the operator‐controlled settings and the subject contrast. HCSR is defined in Ref. 1, as the minimum distance resolvable between two identical point targets, which produces, for a given gain setting, a higher level of backscattering than their surrounding medium, permitting their individual identification. HCSR is usually tested using filaments within a tissue mimicking material embedded in an ultrasound phantom [Fig. 1(b)].

Very few papers have addressed the issue of objective, computer‐based methods for the advanced modes.^10^, ^15^, ^16^ Computer based tests for assessing the difference in image quality of HI vs. B‐mode imaging were published by Van Wijk and Thijssen.^15^ Using a commercial phantom, they found no significant difference in image contrast between the B‐mode and HI mode but they observed an improvement in lateral spatial resolution. Browne et al.^10^ also investigated image quality for the HI and SCI modes using computer‐based tests and a commercial phantom. They found that HI improves lateral spatial resolution and slice thickness as a function of depth but had no effect on contrast resolution and low‐contrast spatial resolution (anechoic target detection). In a follow‐up study,^16^ they used a subcutaneous pig fat layer with a commercial phantom to introduce aberration clutter and saw an improvement in anechoic target detection with HI mode as compared to B‐mode imaging but no improvement in image contrast resolution. In the same study, they also investigated the SCI mode. SCI was found to significantly improve image contrast resolution and anechoic target detection as compared to B‐mode imaging but produced limited improvements in spatial resolution.

## 2 MATERIALS AND METHODS

### 2.A Phantoms

**Figure 2:**
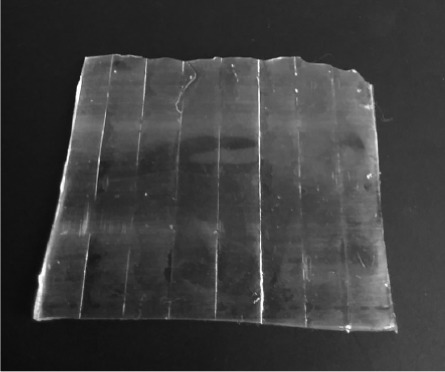
Phase aberration used to test tissue aberration correction mode.

A general‐purpose phantom (Model 040GSE, CIRS, Norfolk, VA, USA) and in‐house manufactured aberration layer were used for the tests. The commercial phantom was made of a material (Zerdine, solid elastic water‐based polymer) which mimics the average acoustic properties of soft tissue: average speed of sound (1540 m/s) and attenuation either 0.5 or 0.7 dB cm^−1^ MHz^−1^). Embedded in the Zerdine material, were targets of known depth and size, which were designed to test the probe plus scanner combination performance. The grayscale (contrast) targets were used to determine image contrast [Fig. 1(a)]. They are cylinders of known diameters and location, having known (nominal) contrast, as compared to the surrounding background material, of: −9, −6, −3, 3, 6 dB, and a hyperechoic target (>15 dB), with an accuracy usually no lower than ±1 dB. The background contrast had a range of ±1 dB. The point spread function (PSF) targets were horizontal and vertical filaments of polymeric material (nylon) with nominal diameter 0.1 mm which were employed to determine the spatial resolution. The commercial phantom cannot mimic image degradation observed in images of real tissue. Therefore, we created a phase aberration layer using silicone elastomer 2 (Sylgard 184; Dow Corning) to test the TAC mode. This layer is shown in Fig. 2. Its thickness was made to vary between 0.9 and 2 mm in a sinusoidal pattern. This pattern repeats three times over its length. To create the layer a mold was fabricated which consisted of a 10 cm × 10 cm acrylic tray with removable sides. The lower surface of the mold was fitted with thin plastic pieces that were cut to create an undulating upper surface using a laser cutter. The mold was sprayed with a release agent (CRC Industries) to allow removal of the layer after setting. The base and curing agent of the elastomer were mixed (10:1 ratio) and degassed to remove visible bubbles. The mixture was then slowly poured into the mold to avoid entraining air and allowed to set at room temperature. After setting, the layer was removed by disassembly of the mold. During testing, the layer was placed on top of the commercial imaging phantom with ultrasound gel on the top and bottom of the layer to ensure proper coupling.

### 2.B US equipment and settings

Examples of our QC tests are illustrated with the GE LOGIQ E9 system for SCI, HI, SR modes, and with the Philips iU22 system for the TAC mode. With the LOGIQ E9 ultrasound scanner, we used the 9L‐D probe which is a linear array with bandwidth of 3.33–10.0 MHz, and the convex probe C1‐6‐D with bandwidth 1–6 MHz. With the iU22 system, we employed the high‐frequency linear probe L17‐5 probe with bandwidth 5–17 MHz. Both the contrast targets and the PSF filaments were at first imaged in B mode and then in sequence, each advanced mode was turned on, while keeping the same settings as the ones established for B mode. With the GE system, we chose the prostate preset. This is because among the display maps available for this preset, there is map E, which is closest to linear, assigning the different brightness levels (representing the echo strength of each target) equally throughout the range of pixel values (0–255). With the Philips system, we chose the breast preset and map 5, which is also closest to linear. In addition, we kept the image processing to the minimum. Features such as frame average (or persistence, temporal filter that averages frames together), rejection (selects a level below which echoes will not be amplified) and suppression (suppresses the noise in the image) were kept to zero and line density was kept to four. The output power percentage was kept to 100, and only one focus was chosen with focus width 1. The remaining parameters: depth, frequency, dynamic range, and gain were adjusted for best visualization of the contrast targets and the filaments as summarized in Table 1.

**Table 1:** General test settings

Probe model	GE Logiq E9		Philips iU22
	9L‐D	C1‐6‐D	L17‐5
Frequency (MHz)	9	5	5–17 (Res)
Focus number and depth (cm)	1 and 3.5	1 and 3.0	1 and 2
Depth (cm)	5	7	3.5
Gain	42	56	100%
Dynamic Range/Compression	72	72	70
Scan line density	4	4	N/A^a^
Persistence	Off	Off	Off
Rejection/Frame Averaging	Off	Off	Off
Noise suppression	Off	Off	Off
Post‐processing (Map)	E	E	5
Test	B, SCI, HI, SR	B, SCI, HI, SR	B, TAC

a This quantity is adjusted automatically in the Philips iU22.

### 2.C Data analysis

#### 2.C.1 Grayscale mapping function and image contrast

**Figure 3:**
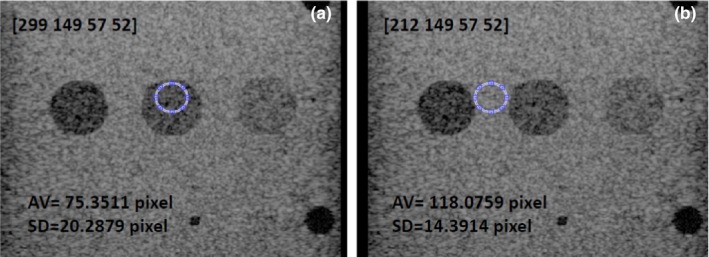
Contrast targets imaged in HI mode. (a) Ellipsoid ROI of size 57 × 52 pixels inside the −6 dB nominal contrast target. (b) ROI of the same size in the adjacent background. In the images, the ROI location and the average and standard deviation of the pixel values inside the ROI is shown.

For each probe‐scanner combination, a number of images of each contrast target were acquired. The DICOM images were imported and read with MATLAB R2014a Image Processing Toolbox (MathWorks, Inc., Natick, MA, USA). An ellipsoid region of interest (ROI) was drawn inside each contrast target using our MATLAB codes and functions available in the Image Processing Toolbox [Fig. 3(a)]. The choice of an ellipsoid shape was somewhat arbitrary and was chosen for convivence. However, we chose the ROI consistently: the same ROI size and shape at the same depth and distance from the target boundary for all the contrast targets imaged. For the linear probe, we chose an ellipse of size 62 × 52 pixels, at a distance from the target upper‐boundary of 8 ± 2 pixels. The uncertainty of ±2 pixels considers the uncertainty in the boundary location. We checked that this uncertainty does not significantly affect the estimation of means and SDs. For the convex probe, we chose a smaller ellipse of size 38 × 27 pixels as the contrast targets appear smaller when viewed with the convex probe. The mean (*E*_*i*_) and the standard deviation (*σ*_*i*_) of the grayscale values inside the ellipsoid ROI was then determined using our MATLAB codes together with functions available in the Image Processing Toolbox [Fig. 3(a)]. For each contrast target and mode, *E*_*i*_ and *σ*_*i*_ were then weighted to obtain the weighted mean and weighted standard deviation (SD)^30^

(1) $$\left\langle E_{T}\right\rangle =\frac{\sum_{i}w_{i}E_{i}}{\sum_{i}w_{i}}$$

with $w_{i}=\frac{1}{\sigma_{i}^{2}}$. The weighted standard deviation (SD) for 〈*E*_*T*_〉 is

(2) $$\sigma_{\left\langle E_{T}\right\rangle }=\frac{1}{\sqrt{\sum_{i}w_{i}}}$$

**Figure 4:**
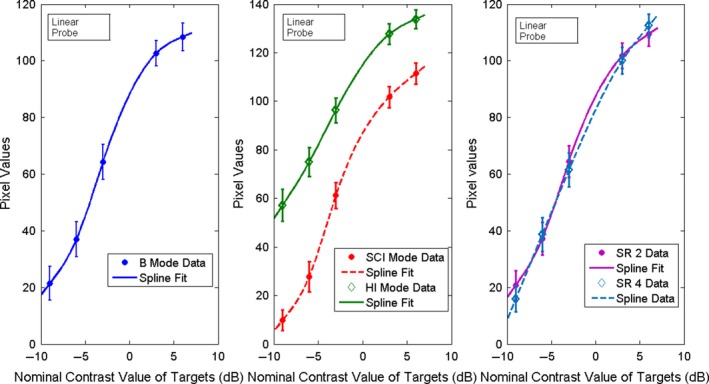
Spline fitting curve fitting of the weighted mean and SD of the pixel values for the contrast targets with nominal contrast: −9,−6,−3,3,6 dB for B, SCI, HI, SR 2, SR 4 modes, for the linear probe.

**Figure 5:**
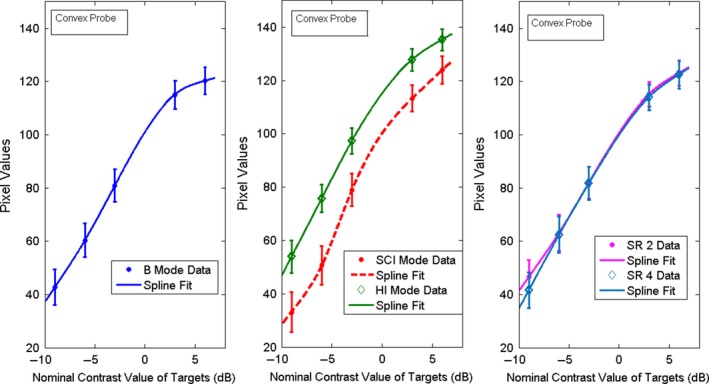
Spline curve fitting of the weighted mean and SD of the pixel values for the contrast targets with nominal contrast: −9,−6,−3,3,6 dB for B, SCI, HI, SR2, SR 4 modes, for the convex probe.

**Figure 6:**
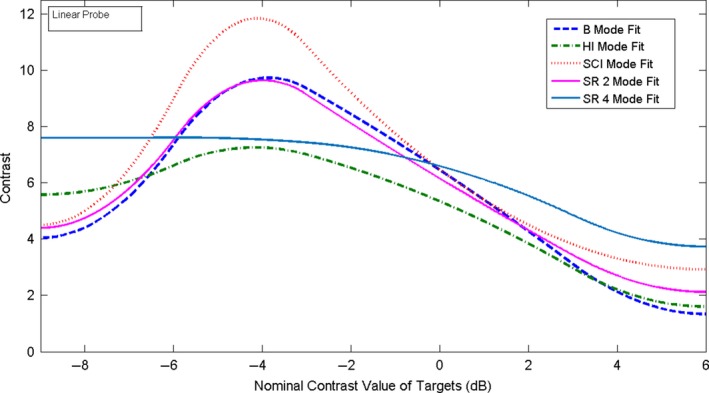
The slope (contrast) of the GMF curves shown in Fig. 4 for the linear probe.

**Figure 7:**
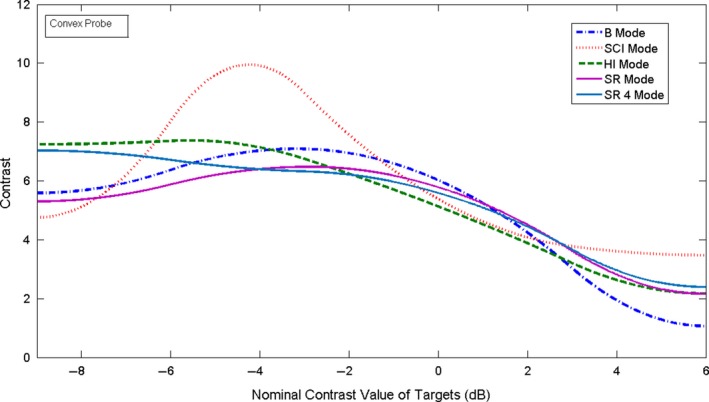
The slope (contrast) of the GMF curves shown in Fig. 5 for the convex probe.

The weighted mean and weighted SD values of the pixel values, 〈*E*_*T*_〉 and $\sigma_{\left\langle E_{T}\right\rangle }$, were then plotted as a function of the nominal contrast value in dB of each target and illustrative examples are shown in Figs. 4 and 5. Curve fitting of the weighted mean and weighted SD values of the pixel values was performed using MATLAB curve fitting toolbox. As may be seen in the figures, the relationship between the pixel values and the echo signal values, as estimated by the nominal contrast value of the targets, is a non‐linear function. This should be expected since compression and digitization of the echo signal (usually to 8 bits) is a nonlinear process. Non‐linear fitting using spline is the best fit for these data. This non‐linear fitting of the data defines the grayscale mapping function (GMF). The GMF curve has a toe, a linear portion, and a shoulder,^1^, ^31^ which are clearly seen in Figs. 4 and 5. The toe and the shoulder of the GMF curve are saturation regions: weak or large echoes produce very little change in the grayscale values, and therefore represent areas of low contrast in the image. In the linear portion, a small change in the echo amplitudes induces a visible change in the grayscale values, which corresponds to the diagnostically useful values as they produce the largest contrast.^8^ The features of the GMF curves are further emphasized by their gradient (slope), which provides a measure of image contrast, as it may be seen in Figs. 6 and 7. These plots suggest that the saturation regions have lower contrast and the contrast is highest in the most linear portion of the GMF curves.

#### 2.C.2 Contrast‐to‐noise ratio

**Figure 8:**
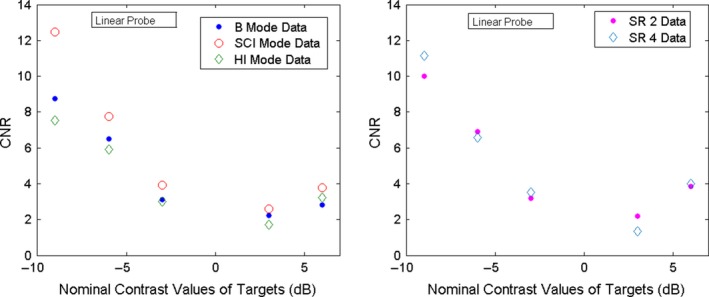
CNR for the linear probe for the contrast targets located at a depth of 3 cm and nominal contrast: −9,−6,−3,3,6 dB.

**Figure 9:**
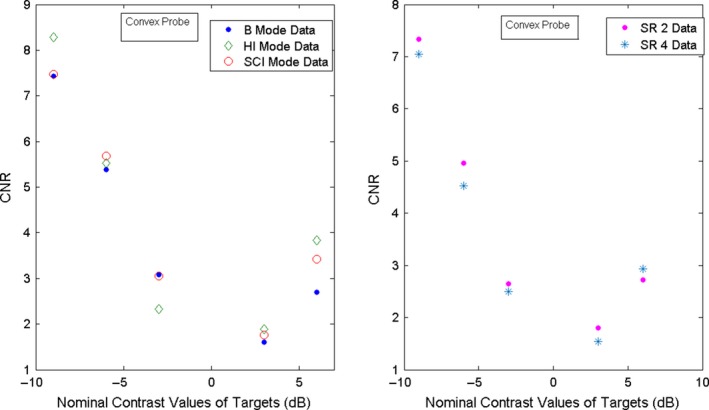
CNR for the convex probe for the contrast targets located at depth of 3 cm and nominal contrast: −9,−6,−3,3,6 dB.

Another important parameter of performance is the Contrast‐to‐noise ratio (CNR)^8^, ^32^

(3) $$CNR=\frac{\left|\left\langle E_{T}\right\rangle -\left\langle E_{B}\right\rangle \right|}{\sqrt{\sigma_{\left\langle E_{T}\right\rangle }^{2}+\sigma_{\left\langle E_{B}\right\rangle }^{2}}}$$

where $\left\langle E_{T}\right\rangle$and $\sigma_{\left\langle E_{T}\right\rangle }$ are the weighted mean and SD for the target as defined in Eqs. (1) and (2), $\left\langle E_{B}\right\rangle$is the weighted mean of a region of background in the phantom of same size and depth as the target ROI, and $\sigma_{\left\langle E_{B}\right\rangle }$ is the corresponding weighted SD. Special care has to be taken to select the background ROI. This is because the background weighted mean $\left\langle E_{B}\right\rangle$ changes significantly from the center location of the image to the border. For best accuracy, it is recommended to choose the background ROI next to the target.^1^ Since there is not enough background space between targets in the phantom, we selected a ROI for both target and background a bit smaller than the one we selected for contrast determination. For the linear probe, we have chosen an ellipse of size 57 × 52 pixels. An example is shown in Fig. 3(b). The CNR data were then plotted as a function of the nominal contrast of the targets and examples are shown in Figs. 8 and 9. Curve fitting was not implemented in these figures, so the CNR is given only for the measured data, which were obtained with a limited set of measurements (10 images for each mode). Therefore, the obtained CNR has limited statistics validity.

#### 2.C.3 High‐contrast spatial resolution

**Figure 10:**
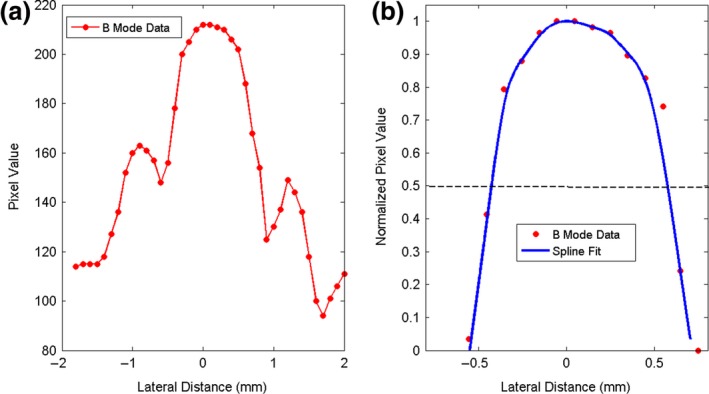
(a) Pixel values of the line passing through the filament highest pixel value as a function of lateral distance. (b) Spline curve fitting of the main peak normalized to one. The position of the maximum pixel was chosen as the origin for the horizontal axis. The filament was imaged with the convex probe operated in B mode.

**Figure 11:**
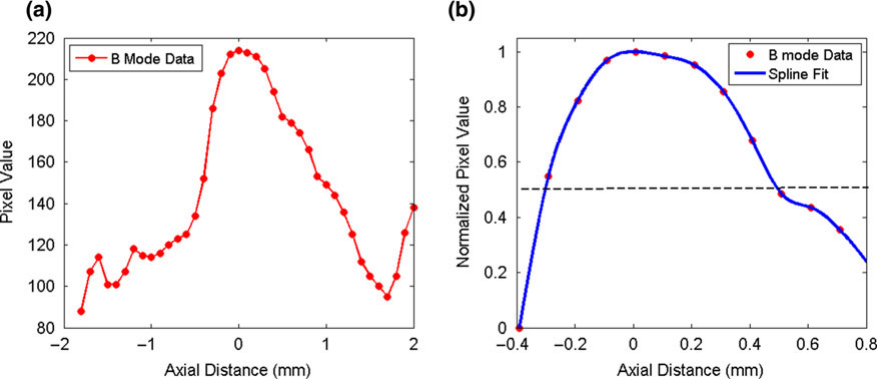
(a) Pixel values of the line passing through the filament pixel highest value as a function of axial distance. (b) Spline curve fitting of the main peak normalized to one. The position of the maximum pixel was chosen as the origin for the horizontal axis. The filament was imaged with the convex probe operated in B mode.

**Figure 12:**
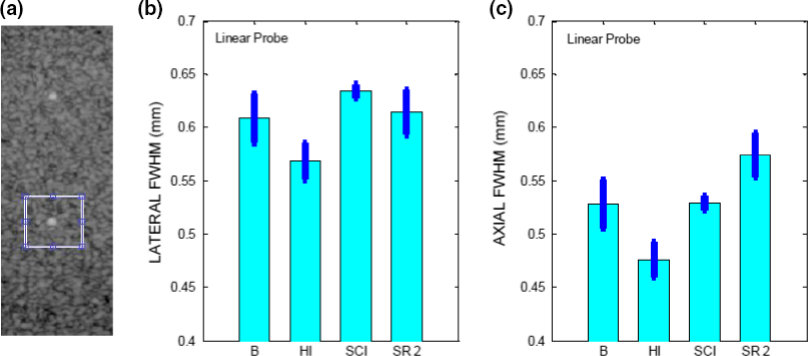
(a) ROI surrounding the filament located at a depth of 2 cm, imaged in HI mode. FWHM (Mean ± SD) along (b) lateral and (c) axial direction for the linear probe operated in B, HI, SCI, SR 2 modes.

**Figure 13:**
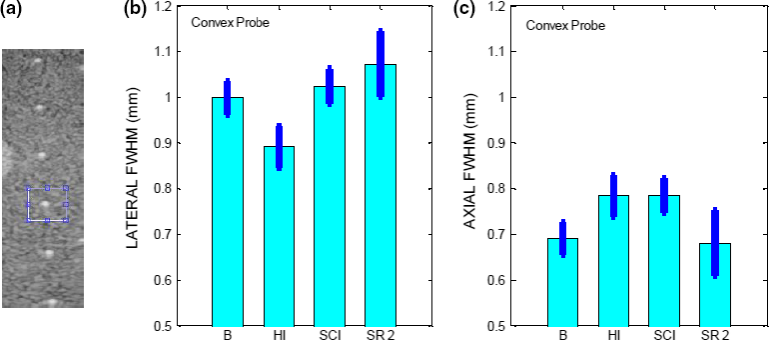
(a) ROI surrounding the filament located at a depth of 4 cm, imaged in HI mode. FWHM (Mean ± SD) along (b) lateral (c) axial direction for the convex probe operated in B, HI, CI, SR 2 modes.

The HCSR is determined in the scan plane along the beam propagation direction z (axial resolution) and in the transversal direction × (lateral resolution) for each mode. An objective method for determining the HCSR consists in determining the FWHM (full width at half maximum) along the lateral (horizontal) and axial (vertical) directions of a given filament^8^, ^9^, ^33^ as explained below. It is recommended to select the filament closest to the elevation focus with the scan and elevation foci coinciding.^6^, ^8^ This is because when the two foci coincide, the sensitivity of the US imaging system is at its highest level. Images of the chosen filament were acquired a number of times (at least five) for each mode. These images were then analyzed using MATLAB Image Processing Toolbox and our MATLAB codes. For each image, a ROI containing the chosen filament and surroundings was chosen. The filament highest pixel value was determined and the lateral and axial pixel lines passing through it were selected. If two highest pixel values and occasionally a few more were present in the ROI, lateral and axial pixel lines passing through each of them were evaluated. Of these lines, the lateral and axial lines selected were the ones whose sum of pixel values was the highest. Representative plots of the pixel values of these lines may be seen in Fig. 10(a) for the lateral direction and Fig. 11(a) for the axial direction. In these figures, the pixel values are plotted as a function of distance in mm. Owing to the presence of side peaks, special care has to be applied to isolate the main peak. For each direction, our method consists in subtracting a suitable background pixel value to the filament pixel values. This background pixel value was chosen by analyzing the background surrounding the filament for each image. Curve‐fitting was implemented on these values using spline. The maximum of fitted curve was determined and the curve was normalized with respect to this maximum value. Illustrative examples of this procedure are shown in Fig. 10(b) for the lateral direction and in Fig. 11(b) for the axial direction. The x‐coordinates in mm of the two points (x_1_ and x_2_) having y‐coordinates 0.5 were determined. For each mode, the FWHM was then averaged. Illustrative examples are shown in Figs. 12 and 13.

**Figure 14:**
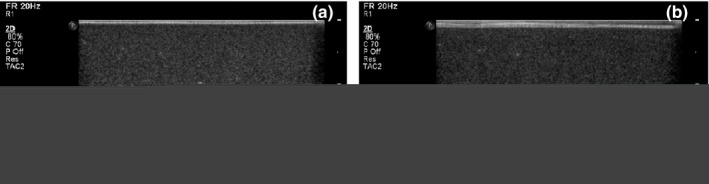
(a) Image of the filament located in a depth of 2 cm acquired with the high‐frequency probe in TAC mode. (b) Image of the same filament acquired with the high‐frequency probe in TAC mode and the layer on top of the phantom.

**Figure 15:**
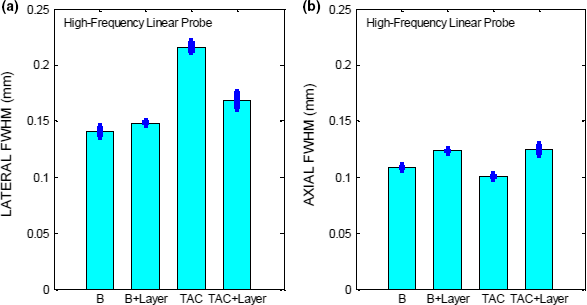
FWHM along (a) lateral (b) axial direction for the linear high‐frequency probe operated in B, and TAC modes for the filament at depth 2 cm.

The HCSR test was also performed with the high‐frequency probe operated in B and in TAC modes without and then with the aberration layer on top of the commercial phantom. In B mode, the image (not shown) of the chosen filament was of high quality. This is because the speed of sound in the phantom is the same as the one assumed by the US system and the electronic signals induced by the echo from the filament emerge from the delay‐lines of the probe at identical times and in phase, producing a good image of the filament. The aberration layer was then placed on top of the phantom and images of the filament were also taken in B mode. The presence of the layer generates random time delays, *Δt*, which are related to the variation in thickness of the layer as (for an example, see Ref. 34)

(4) $$\Delta t\;\approx \;D\cdot (\frac{1}{c}-\frac{1}{c_{\mathrm{layer}}})$$

where *D* is the layer thickness, *c* = 1540 m/s and *c*_*layer*_ is the speed of sound in the layer, which was not measured in our experiments. The TAC 2 mode was then turned on and images of the filament, were taken without [Fig. 14(a)] and with [Fig. 14(b)] the layer which appeared defocused and distorted. The filament appeared defocused and distorted. This arises because the TAC 2 mode assumes a different speed of sound than the one in phantom and the delay‐lines are unable to provide phase coherence for the returning echo. The FWHM was determined in B and TAC modes with and without the layer and the results are illustrated in Fig. 15.

## 3 RESULTS

### 3.A Grayscale mapping function and image contrast

For evaluating the GMF and the image contrast, we imaged the contrast targets located at a depth of 3 cm. The weighted mean and weighted SD of the pixel values, as defined by Eqs. (1) and (2), were obtained from ten images. The results are plotted as function of the nominal contrast of each target in Fig. 4 for the linear probe 9L and in Fig. 5 for the convex probe C1‐6. As it may be seen, the linear portion of the GMF curve is primarily associated with the targets of negative (hypoechoic) contrast for both probes and modes. This simply confirms what can be deduced by visual inspection of the images that shows clearly that at the chosen settings, the negative contrast targets are better visualized than the positive (hyperechoic) ones. As may be deduced from the Figs. 4 and 5, each probe‐mode combination has a characteristic GMF curve with saturation regions and linear regions. The gradient of the GMF curves shown in the Figs. 4 and 5 may be seen in Fig 6 for the linear probe and in Fig. 7 for the convex one. The plots for the linear probe suggest that the saturation regions have lower contrast and the contrast is highest in the most linear portion of the GMF curves, with SCI mode having the highest contrast.

For the convex probe, one may see the same trends, although the data are noisier, having larger SD. The contrast is reduced in the saturation region of the curve (+6 dB target region) for all the modes and the −9 dB region of the curve is not a saturation region except for the SCI mode. An average slope can also be defined in the most linear region of each GMF curve. For example, for the linear probe the most linear region for all the GMF curves is between about −6 and 0 dB, and the average slope is respectively for SCI, B, SR2, SR4, and HI mode: 9.83, 8.54, 8.34, 7.32, 6.66 pixel/dB.

### 3.B CNR

The CNR (Eq. (3)) is shown in Fig. 8 for the linear probe and in Fig. 9 for the convex probe. For both the linear and convex probes, CNR is better for the negative contrast targets than the positive ones for all the modes, with the worst CNR for +3 dB target. This is clearly visible with the naked eye and the CNR analysis reproduces it correctly. The comparison among modes is however limited by low statistics. Nevertheless, the CNR plots suggest the following. For the linear probe, SCI mode has a better CNR, especially for the negative contrast targets. The CNR for the convex probe is worse than for the linear probe. As may be seen in Fig. 9, the CNR values for the convex probe are closer than the corresponding ones for the linear probe for all the modes. The HI mode has a somewhat better CNR than SCI except in the region where contrast is highest for SCI mode.

### 3.C HCSR

For the HCSR test, it is recommended to select the filament closest to the elevation focus. However, since the depth of the elevation focus was not established at acceptance testing for our probes, we have chosen for the linear probe, the vertical filament located at the depth of about 2 cm and for the convex probe, the one located at the depth of about 4 cm. For each mode, images of the chosen filament were acquired five times for the linear probe and ten times for the convex probe as the data were noisier. The filament highest pixel value was determined and the lateral and axial pixel lines passing through it were selected as described in Section 2.C.3. Representative plots of the pixel values for these lines may be seen in Fig. 10(a) for the lateral direction and Fig. 11(a) for the axial direction. In these figures, the pixel values are plotted as a function of distance in mm, obtained by the knowledge of the pixel size and the number of pixels along each line. The pixel size for the linear probe was estimated to be 0.05 mm/pixel and for the convex probe 0.1 mm/pixel. The FWHM which provides an estimate of the HCSR was determined as described in Section 2.C.3. For each mode, the FWHM was averaged and the results for the average ±1 SD, are shown in Fig. 12 for the linear probe and in Fig. 13 for the convex probe. These results were obtained with a small number of images. Nevertheless, Figs. 12 and 13 suggest that the axial HCSR is better than the lateral one. For the linear probe, the results along the lateral direction are nosier than along the axial one, and HI mode performs better than all the other modes, which have a very similar performance. The HCSR for the convex probe is worse than for the linear probe. The performance of each mode is very similar for this probe, with HI mode performing slightly better on average along the lateral direction. No significant difference is seen along the axial direction among the modes, probably as the result of noise.

### 3.D Tissue aberration correction mode

For evaluating the TAC mode, we performed the HCSR test using the Philips iU22 scanner with the high‐frequency linear probe L17‐5. The pixel size was estimated to be 0.03 mm. As described earlier, the image processing settings were kept to the minimum. Five images of the filament located at a depth of 2 cm were acquired in B and TAC modes with and without the aberration layer. The results for the FWHM are summarized in Fig. 15. At first, filament images were obtained in B mode without the aberration layer and then with the layer. Owing to the high frequency of the probe, the resolving power of the US system is high, as may be seen in Fig. 15(b), where the FWHM along the axial direction is close to the actual size of the filament, which is 0.1 mm. Then, the layer was placed on top of the phantom with ultrasound gel on the top and bottom of the layer to ensure the best coupling; care was taken to avoid trapped air bubbles between the layer and phantom. As may be seen in Fig. 15, the presence of the layer only slightly increases the FWHM in the axial and lateral directions, with the effect being stronger in the axial direction than in the lateral one. Next, the TAC 2 mode was turned on and the filament image appeared strongly distorted with the image of the filament cross‐section squeezed along the axial direction and enlarged along the lateral direction [Fig. 14(a)]. As a result, the FWHM along the axial direction is smaller than the corresponding one in B mode and the FWHM in the lateral direction is larger. Then, the layer was placed on top of phantom. The presence of the layer, although still defocuses the filament image, corrects somewhat the filament distortion [Fig. 14(b)]. This may also be seen in Fig. 15, where the FWHM along the axial direction is increased, correcting in part for the squeezing of the filament, and along the axial direction the FWHM is decreased. This effect may arise because the speed of sound in the layer is closer in magnitude to the speed of sound assumed in the TAC 2 mode.

## 4 DISCUSSION

We have presented computer‐based methods for estimating the following parameters of image quality: GMF, image contrast, CNR, and HCSR. Provided that the targets are imaged using the same settings with minimum processing and the scanning conditions are kept as similar as possible (for example target always at the image center), then these parameters may provide an estimate of the performance of a given mode for the probe plus scanner combination evaluated. They could be also useful in developing standardized methods for evaluating the advanced ultrasound imaging modes and for comparing the performance of scanner plus probe combination with one another one and against the standard specifications provided by the manufacturer.

The examples provided in this study are aimed to illustrate our computer‐based QA method. The preliminary results of these examples suggest that the GMF for the linear probe is a sigmoid curve with the highest contrast in the most linear portion of the curve. This remains true also for the convex probe; however, for this probe the linear portion of the GMF is greater. The reason for this difference might be associated with different probe design, manufacturing, scanning format and settings. For the linear probe, CNR is best for SCI and very similar for all the other modes. For the convex probe, CNR is very similar for all the modes as the data are much noisier. The data for HCSR show that the axial HCSR is better than the lateral one. For the lower‐frequency linear probe, HI mode performs better than all the other modes, which have a very similar performance. The HCSR for the convex probe is worse than for the linear probe. The performance of each mode is very similar for this probe, with HI mode performing better on average along the lateral direction. For the higher‐frequency linear probe, we have considered B mode and TAC mode (speed‐of‐sound correction mode) and made HCSR measurements. This probe is extremely sensitive and can detect very small variations in speed of sound, although the imaging system is not capable to adjust the speed of sound adaptively, according to the speed of sound in material. Adaptively adjusting speed of sound is a difficult problem that still waits for a definitive solution.

The above results suggest that the selected parameters can be useful in a quality assessment protocol of HI, SCI, SR, and TAC due to their sensitivity to ultrasound image characteristics and distortions. Moreover, a phase aberration layer could be used in addition to commercial ultrasound phantoms for testing how well the ultrasound system corrects for artifacts. The goodness of the system may be related to the characteristics and number of aberration layers whose overall distortion effect cannot be properly compensated in the diagnostic image. To this aim, a threshold on the tolerable distortion may be established. In this regard, future studies can be focused on the design and the development of a set of standardized phase aberration layer to be applied on a commercial ultrasound phantom. Nevertheless, addition studies are required in order to determine the reliability and robustness of the proposed QA method. For example, we know that the above parameters of performance are affected by the scanning conditions and for this reason we kept these conditions as similar as possible. However, we have not investigated in a systematic way to which degree the scanning conditions affect our results. It also important to investigate the inter‐observer reproducibility and this aspect was not considered at all in this study. In addition, the results of this paper are based on a few measurements (5–10 for each mode) and for improving the statistics, a feasible option is to automate data analysis so that many more images for each mode can be quickly analyzed. Analyzing many images using automation could be also useful in reducing the effect of variability associated with slightly different scanning conditions and the effect of inter‐observer variability.

## 5 CONCLUSIONS

Performances of advanced US imaging modes currently available in US imaging systems such as HI, SCI, adaptive SR, and TAC modes, can be quantified by evaluating parameters of image quality, such as GMF, image contrast, CNR, and HCSR. In this paper, a first evaluation of the aforementioned parameters is proposed and some preliminary image analysis have been shown without and with a phase aberration layer applied on the scanning window of a commercial ultrasound phantom. Despite the limitations of this study, some general conclusions can be drawn: the adaption and application of B‐mode image quality parameters to ultrasound advanced imaging is promising and feasible due to their good sensitivity to the ultrasound image characteristics and because they are well known in the scientific community. Moreover, a cheap and user‐friendly tool such as a phase aberration layer, may be used in addition to other common test objects, i.e., commercial ultrasound phantoms, for testing the ultrasound system capability to correct for artifacts. This may be useful to clinical medical physicists and technicians in testing the performance of US imaging systems before new algorithm or acquisition modes are employed in clinics.

## CONFLICTS OF INTEREST

No Conflict of interest.
